# Technical recommendations for clinical translation of renal MRI: a consensus project of the Cooperation in Science and Technology Action PARENCHIMA

**DOI:** 10.1007/s10334-019-00784-w

**Published:** 2019-10-18

**Authors:** Iosif Mendichovszky, Pim Pullens, Ilona Dekkers, Fabio Nery, Octavia Bane, Andreas Pohlmann, Anneloes de Boer, Alexandra Ljimani, Aghogho Odudu, Charlotte Buchanan, Kanishka Sharma, Christoffer Laustsen, Anita Harteveld, Xavier Golay, Ivan Pedrosa, David Alsop, Sean Fain, Anna Caroli, Pottumarthi Prasad, Susan Francis, Eric Sigmund, Maria Fernández‐Seara, Steven Sourbron

**Affiliations:** 1 Department of Radiology, Addenbrooke’s Hospital, Cambridge University Hospitals NHS Foundation Trust, Cambridge, UK; 2 Department of Radiology, Ghent University Hospital and Ghent Institute for Functional and Metabolic Imaging, Ghent University, Ghent, Belgium; 3 Department of Radiology, Leiden University Medical Center, Leiden, The Netherlands; 4 Developmental Imaging and Biophysics Section, UCL Great Ormond Street Institute of Child Health, London, UK; 5 Translational and Molecular Imaging Institute and Department of Radiology, Icahn School of Medicine at Mount Sinai, New York, NY USA; 6 Berlin Ultrahigh Field Facility, Max Delbrueck Center for Molecular Medicine in the Helmholtz Association, Berlin, Germany; 7 Department of Radiology, University Medical Center Utrecht, Utrecht University, Utrecht, The Netherlands; 8 Department of Diagnostic and Interventional Radiology, Medical Faculty, University Dusseldorf, 40225 Dusseldorf, Germany; 9 Division of Cardiovascular Sciences, School of Medical Sciences, Faculty of Biology, Medicine and Health, University of Manchester, Manchester, UK; 10 Sir Peter Mansfield Imaging Centre, University of Nottingham, University Park, Nottingham, UK; 11 Imaging Biomarkers Group, Department of Biomedical Imaging Sciences, University of Leeds, Leeds, UK; 12 Department of Clinical Medicine, MR Research Centre, Aarhus University, Aarhus, Denmark; 13 Brain Repair and Rehabilitation, Institute of Neurology, University College London, London, UK; 14 Department of Radiology, Advanced Imaging Research Center, University of Texas Southwestern Medical Center, Dallas, USA; 15 Department of Radiology, Beth Israel Deaconess Medical Center, Harvard Medical School, Boston, MA USA; 16 Departments of Biomedical Engineering, Radiology, and Medical Physics, University of Wisconsin, Madison, WI USA; 17 Department of Biomedical Engineering, Istituto di Ricerche Farmacologiche Mario Negri IRCCS, Bergamo, Italy; 18 Department of Radiology, Center for Advanced MR Research, NorthShore University Health System, Evanston, IL USA; 19 Department of Radiology, Center for Biomedical Imaging (CBI), Center for Advanced Imaging Innovation and Research (CAI2R), NYU Langone Health, New York, NY USA; 20 Department of Radiology, Clínica Universidad de Navarra, Pamplona, Spain

**Keywords:** Kidney, Imaging, Biomarkers, Standardisation, Consensus

## Abstract

**Purpose:**

The potential of renal MRI biomarkers has been increasingly recognised, but clinical translation requires more standardisation. The PARENCHIMA consensus project aims to develop and apply a process for generating technical recommendations on renal MRI.

**Methods:**

A task force was formed in July 2018 focused on five methods. A draft process for attaining consensus was distributed publicly for consultation and finalised at an open meeting (Prague, October 2018). Four expert panels completed surveys between October 2018 and March 2019, discussed results and refined the surveys at a face-to-face meeting (Aarhus, March 2019) and completed a second round (May 2019).

**Results:**

A seven-stage process was defined: (1) formation of expert panels; (2) definition of the context of use; (3) literature review; (4) collection and comparison of MRI protocols; (5) consensus generation by an approximate Delphi method; (6) reporting of results in vendor-neutral and vendor-specific terms; (7) ongoing review and updating. Application of the process resulted in 166 consensus statements.

**Conclusion:**

The process generated meaningful technical recommendations across very different MRI methods, while allowing for improvement and refinement as open issues are resolved. The results are likely to be widely supported by the renal MRI community and thereby promote more harmonisation.

## Introduction

**Fig. 1:**
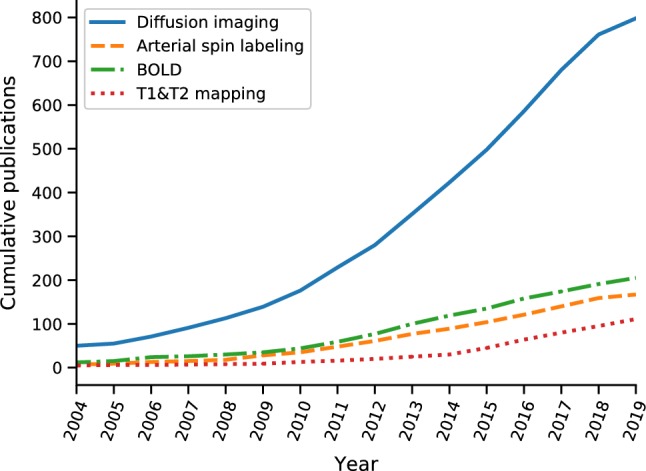
Evolution in the cumulative number of publications in the context of renal imaging for each class of techniques for which recommendations were developed. Data were retrieved from multiple (*n* = 124) PubMed searches using the string: (kidney* OR renal) AND < TECHNIQUE > AND MRI AND < year > [PDAT], where < TECHNIQUE > is one of the following substrings: (diffusion-weighted imaging OR diffusion tensor imaging OR intravoxel incoherent motion), arterial spin label*, blood oxygenation-level dependent and (*T*_1_ mapping OR *T*_2_ mapping), respectively, for the techniques: diffusion imaging, arterial spin labelling, BOLD and T_*1*_&T_*2*_ mapping and < year > varied from 1989 to 2019 with increments of 1 (only the last 15 years shown). Duplicates were removed. This plot is meant to capture the overall trend over time rather than accurate numbers of publications and the search could be refined by including for instance abbreviated names of techniques, followed by manual removal of search results that are out of scope

The past few years have seen a surge in the interest in functional and quantitative magnetic resonance imaging (MRI) of the kidney (Fig. 1). In October 2015, the first international meeting on renal MRI was organised in Bordeaux [1], followed in 2017 by the second meeting in Berlin [2], and a third meeting in October 2019 in Nottingham, UK [3]. In 2016, a pan-European network of researchers in renal MRI (PARENCHIMA) was funded for 4 years by the European Cooperation in Science and Technology (COST) [4]. In the UK, a Renal Imaging Network (UKRIN) was set up in 2016 in collaboration with the charity Kidney Research UK [5], and in 2018 was awarded a Medical Research Council (MRC) Partnership grant to develop a national infrastructure for clinical renal MRI research [6]. Also in 2018, the National Institute of Diabetes and Digestive and Kidney Diseases (NIDDK) at the National Institutes of Health in the USA conducted a workshop on renal imaging as a critical review for current state-of-the-art and to plan potential future endeavours [7].

The interest in renal MRI is strongly driven by clinical demand, as evidenced by the leading role of nephrologists and radiologists in developing networks and the increasing exposure of renal MRI in the clinical literature. In 2018, the leading European nephrology journal Nephrology Dialysis Transplantation published a special issue on renal MRI [8], with a clinical position statement supported by over 30 authors including leading European nephrologists [9]. The authors emphasise that renal diseases pose a significant and escalating socioeconomic burden on health-care systems worldwide, and that the development of better diagnostics and prognostics is well recognised as a key strategy to address these challenges. MRI biomarkers show promise in that respect due to their non-invasive nature and potential for early detection of parenchymal changes caused by disease progression or treatment effects.

A key challenge in clinical translation of MRI biomarkers is the need to build strong evidence of their clinical utility on a scale and with a level of rigour that would satisfy the expectations of regulators. This is particularly challenging in chronic kidney disease (CKD) where very large and/or very lengthy studies are required to collect sufficient outcome data. The magnitude of this challenge is illustrated by the qualification by the Food and Drug Administration (FDA) and the European Medicines Agency (EMA) of total kidney volume as a prognostic enrichment biomarker for autosomal dominant polycystic kidney disease (ADPKD)—one of only a handful of clinical biomarkers approved by FDA and the only imaging biomarker so far [10]. The qualification was the end result of a process that lasted over a decade and was only possible after a coordinated effort of the entire ADPKD community in harmonising and combining data from various sources. All other renal MRI biomarkers are at the start of that trajectory [11–14]. Evidence of clinical utility is emerging from small studies, but there is a clear need for larger multi-centre studies.

The first step in scaling up the evidence level is the creation of a more harmonised and standardised approach to data collection. Indeed, MRI sequences are complex and depend on many parameters that must be optimised and fine-tuned separately. Similarly, a wide range of simplifying assumptions can and must be made to arrive at well-determined models during the analysis phase. Currently different groups make different choices of MRI pulse sequences and data analysis steps, and therefore the data are not necessarily directly comparable. Moreover, calibration and quality control of MRI equipment and the resulting measurements are not common practice and not mandatory, unlike imaging modalities that use ionising radiation. The lack of readily available and generally accepted protocols also creates barriers for new groups and clinical sites, often requiring extensive and costly in-house optimisation.

In May 2018, a drive towards standardisation in renal MRI was initiated by the task force on Technical Recommendations for Clinical Renal MRI, of the COST Action PARENCHIMA [4]. The specific aims of the task force were to (1) develop a process for generating technical recommendations across different renal MRI modalities, and (2) apply this in areas of key current interest. For this first stage, it was decided to focus on five classes of renal MRI techniques that were prioritised by the clinical working group in PARENCHIMA [11–14]: renal *T*_1_ and *T*_2_ mapping, arterial spin labelling (ASL), diffusion-weighted imaging (DWI) and blood oxygenation level-dependent MRI (BOLD). Combined, they assured a broad coverage of potential biomarkers with very different pathophysiological origins to test and refine the process before rolling it out more widely across renal MRI methods.

We report in this paper how the process for generating technical recommendations was developed (methods) and describe the process itself in detail (results). The specific technical recommendations for these four renal MRI techniques are reported in four separate publications (submitted).

## Methods

As a basis for discussion, the task force lead (S.S.) drafted a framework and process for development of the technical recommendations throughout May and June 2018. The draft was placed on the public task force website [15] for consultation until the date of the PARENCHIMA plenary meeting in Prague (Czech Republic, October 4th 2018). Feedback was invited by June 24 via the PARENCHIMA mailing list including over 200 experts in renal MRI.

At the same time, four expert panels were created to develop the recommendations for renal *T*_1_ and *T*_2_ mapping, ASL, DWI and BOLD methods. Invitations to join those panels were made to the authors of review papers [11–13] and also through an open invite via the PARENCHIMA online community. A call for experts was also made at the NIDDK renal imaging workshop in July 2018, and corresponding authors from recent publications were contacted individually. All invitations to join the panels included specific requests to feed back on the online draft process. After the panels were populated, the task force lead invited one senior member to act as chair, who then identified a junior co-chair.

As a publication route for the recommendations and the process itself, the task force proposed a special issue on renal MRI biomarkers to the journal Magnetic Resonance Materials in Physics, Biology, and Medicine (MAGMA). The proposal was accepted by the editorial board in September 2018 with a deadline of July 1 2019 for the submission of recommendations.

The draft process was discussed, finalised and agreed upon during the public plenary PARENCHIMA meeting in Prague (October 4 and 5, 2018) during two dedicated 2-h sessions (attendance around 50). The discussion included a review of the draft process and feedback, a presentation by one of the authors of the successful recommendations initiative for ASL in the brain [16], progress reports of the four expert panels, and a group discussion on the draft process. After the meeting, the online draft process was updated to reflect the decisions made in Prague and circulated on October 7, 2018 to all members of the expert panels for final feedback and approval by October 13, 2018.

The agreed process included consensus formation by a Delphi method [17], which involved iterative data collection through surveys. The four expert panels started the implementation on October 13, 2018 and completed a first round of surveys before March 2019. On March 18 and 19, 2019 an intermediate meeting was organised in Aarhus (Denmark) including the task force lead and four to five representatives from each panel [18]. The aim of the meeting was: (1) to evaluate and interpret the results from the first survey and plan the next iteration; (2) to identify discrepancies and overlaps between panels and harmonise the process going forward. After the Aarhus meeting, a final round of surveys was performed, from both the original respondents and additional invitees identified in the literature and within PARENCHIMA.

An ad hoc meeting with the task force lead, the panel chairs, co-chairs and available participants of the panels was organised during the ISMRM (International Society of Magnetic Resonance in Medicine) in May 2019 (Montreal, Canada). At this meeting it was agreed that response numbers and levels of agreement were adequate and it was decided to close the surveys for further submissions. The final reports of the process itself (this paper) and all four technical recommendations were written up independently, and subsequently edited for formatting and presentation as a coherent set of results. After approval by all authors, the wording of the process on the task force website [15] was replaced by a link to a preprint of this paper. A GitHub page was created as a placeholder for future submissions of compliant MRI protocols and released on Zenodo [19].

## Results

We report here the process for developing technical recommendations in clinical MRI agreed upon by PARENCHIMA. This includes a specific aim and three long-term objectives, four guiding principles and an explicit seven-stage process.

The specific aim of the PARENCHIMA consensus process was defined as “the development and maintenance of technical recommendations on the measurement of clinical renal MRI biomarkers that are widely accepted by a broad body of experts in the field”. Three broader, more long-term objectives were defined, underlining the understanding that standardisation of current methods and innovative design of their next iteration are both crucial endeavours to clinical and scientific progress:

1. To promote a more standardised approach to renal MRI biomarker acquisition. This will facilitate direct comparison of quantitative measures by different groups and the evaluation of new technical developments. In addition, this can lead to subsequent clinical trials with nearly identical outcome measures, enabling a rapid establishment of Cochrane type of reviews to change clinical practice.
2. To improve the efficacy of research in renal MRI biomarkers and grow the field by providing a recommended list of parameters to guide the setup of protocols. This will significantly reduce the barriers for setting up new clinical studies, especially when they involve multiple sites and/or vendors.
3. To identify priorities for future research and development by highlighting aspects of quantitative MRI methods where no recommendation is currently possible. These most likely relate to advanced contrast features that are yet to be definitively understood and should remain an active area of innovation and research.

Four general principles were defined to guide the development of the recommendations:

1. *Promoting uptake* Recommendations should be put forward in consensus by a representative expert panel of scientists, using a Delphi method to avoid a bias caused by peer pressure. This will ensure that they are widely supported and maximise their global uptake in future studies. Every effort should be made to invite and include all experts currently active in the relevant field and ensure the panel covers expertise with hardware and software from multiple MRI vendors.
2. *Building on expertise* Ideally, the recommendations should be based on hard scientific evidence that identifies the “best” measurement approach for any specific MRI biomarker. In practice, the evidence will often be insufficient, and in that case the recommendations can be based on the personal expertise of the panel members. In cases where a consensus cannot be reached, the panel can instead flag the issue as a priority for future research and development.
3. *Promoting innovation* The technology of MRI is rapidly evolving and new insights and data are emerging on a regular basis. Recommendations should not act as a brake on innovation, but rather promote it by offering a well-accepted benchmark for new developments. Recommendations should be version controlled and revisited by the panel at regular intervals, or ad hoc when new evidence and technology become available.
4. *Serving the context of use* The optimal approach may depend on the clinical application (e.g. native vs. transplanted kidneys, children vs. adults) and may also be biomarker specific (e.g. fractional anisotropy vs. apparent diffusion coefficient). Other constraints may exist, such as maximum duration or cost of the scan. Where appropriate, panels should therefore consider dedicated recommendations for individual clinical application areas.

A seven-stage process was defined to develop the recommendations, covering the full life cycle from convening the panel to long-term maintenance:

- *Stage 1. Composition of the panels* Convene a panel of experts that is representative of the field, including experts from across the world and ideally covering expertise with all major vendors. Identify a senior chair and a junior co-chair to lead the panel. Their role is to ensure the membership of the panel is representative of the field, coordinate the development of the recommendations, ensure compliance with the general principles and agreed processes, ensure timely delivery, and act as lead authors on the ensuing publications.
- *Stage 2. Definition of the method and context of use(s)* Identify which specific MRI biomarkers or MRI method will form the subject of the technical recommendations, and for which clinical questions they are to be used. The definition can be updated dynamically as the process develops, for instance if first results demonstrate that the original scope was too narrow, too wide, or too open for interpretation.
- *Stage 3. Review of literature* Perform a detailed review of recent literature to identify key issues and inform the content of the first round of surveys. Extract technical parameters, tabulate and compare them to identify key areas of disagreement. Contact authors for missing technical specification. If relevant, compare reported biomarker values against technical parameters across the literature to identify the limiting factors.
- *Stage 4. Review of technical protocols* Contact panel members and authors of recent literature with a request to share study protocols (including patient preparation and image processing) and vendor-specific MRI acquisition protocols. Review and compare these protocols to identify differences in parameters that have not been reported and gain a better understanding of more intricate differences between vendors, scanner models and software versions. This could include a review of data on different MR systems using reference objects or phantoms.
- *Stage 5. Delphi consensus formation* Generate consensus statements using an approximation to the two-step modified Delphi method [17] to ensure all opinions are heard free from peer pressure.
  The Delphi method is very well suited to reduce the bias caused by dominant personalities influencing the opinion of the group. It is an iterative method that determines reliable consensus in practice guidelines on health-care-related issues [20, 21] and on topics where there is little or no definitive evidence and where opinion important [22]. At each iteration, participants are invited to respond to a survey that will also include an anonymous summary of the previous responses. Discussion in a face-to-face meeting between all respondents usually follows one or several of the rounds.
  In the approximate Delphi method adopted by PARENCHIMA, consensus on a topic is pre-defined as at least 75% agreement. At least two rounds of surveys must be completed. Statements may be rephrased after each iteration, and additional respondents can be recruited. The first survey has an open format where the panel defines questions that address the areas of disagreement identified in stage 3 and 4 in a format most appropriate to the question. Examples are multiple-choice questions for optimal values of a technical parameter, or a binary agree/disagree format for qualitative statements. The survey in subsequent rounds must have a simplified multiple-choice format with only three options: (1) “I agree”, (2) “I disagree”, (3) “I have insufficient experience to make a recommendation”. There must be an open comment box after each question to explain the choice that was made. Questions that reach consensus in a previous round will be closed for voting, but are summarised to the respondents in the subsequent round, with an opportunity to comment in a free-text field.
  Consensus statements may refer to all different areas relevant to an MRI biomarker: patient preparation, sequence details (acquisition), quantification model, data analysis and reporting. To interpret the results, the surveys should collect relevant data on the background of the responder (e.g. expertise, level of experience). For responders that work as a team or in close collaboration, each individual team member can submit a separate response to the surveys provided they are answered independently and without discussing the questions or answers. The chair and the co-chair of the expert panels can also submit responses. Survey respondents should be instructed to select the answer that reflects their personal opinion and not necessarily their current practice (which may be limited due to available infrastructure).
  A face-to-face meeting is held after the first round of surveys with a representative cross section of panel members. The purpose of this meeting is to refine and extend the surveys for future rounds and drive consensus on contentious issues by careful consideration of the arguments.
- *Stage 6. Reporting of recommendations* The recommendations should be published in a peer-reviewed journal using vendor-neutral language and terminology. These should detail not only the recommendations themselves, but also the process and rationale underlying the choices that were made to help experts understand their scope and limitations.
  Where possible, detailed vendor-specific implementations in compliance with the recommendations should be published as supplementary material on a version-controlled open-access website. If the recommended approach is not available in particular scanner models or software versions, the panel may instead provide some guidance on how to set up acceptable alternative sequences.
  For reporting of results, a “traffic light” system is proposed to issue recommendations based on the degree of consensus achieved through the survey. A “green light” in a question indicates consensus (closed issues). An “orange light” indicates an open issue where responses show clear preferences and a consensus is within reach. A “red light” is an open issue where no clear recommendation emerges and more info and data are needed. When calculating the percentage of responses, the abstain responses (i.e. “I have insufficient experience to make a recommendation”) will be excluded. However, the percentage of abstain responses for each item will be reported, to reflect the level of familiarity of the experts with the topic and to assess the current level of interest in the topic within the community.
  The authorship on the publications reporting the recommendations will be defined according to the ICMJE criteria (International Committee of Medical Journal Editors). In particular, this means that authors have provided *substantial* contributions to the development of the recommendation. Those who wish to express their support to the recommendations without having contributed substantially can be recognised as signatories in an acknowledgement section. There are various ways that the panel members can contribute substantially to the process:
  1. Contributing technical details of protocols or responses to surveys.
  2. Helping to collect, tabulate and compare protocols and surveys submitted.
  3. Helping to develop, organise and document the online supplementary material (GitHub or equivalent).
  4. Regularly taking part in teleconferences or discussions of the panel to define a consensus.
  5. Other substantial contributions to the conception or design of the work; or the acquisition, analysis, or interpretation of data for the work (specify).
  6. Drafting the work.
  7. Revising the work critically for important intellectual content.
  8. Final approval of the version to be published.
  9. Agreement to be accountable for all aspects of the work in ensuring that questions related to the accuracy or integrity of any part of the work are appropriately investigated and resolved.
  To qualify for authorship, a panel member will therefore need at least one contribution from 1 to 5 AND at least one contribution from 6 to 7 AND 8 AND 9. Authorship order will align to the following principles: first author: junior co-chair; last author: senior chair; “middle authors”: filled in the survey but no other contributions; “outer authors”: filled in the survey and helped organise the material (junior at the front, senior at the end).
- *Stage 7. Maintenance of the recommendations* The panel will remain in existence, though its membership may evolve. At regular intervals or on an ad hoc basis, the panel will revisit the recommendations, review novel evidence and formulate an upgrade if needed. An upgrade can consist of changing the traffic light of any given recommendation (e.g. orange to green), adding new consensus statements or refining existing statements (e.g. narrower range of parameters). Upgrades should be decided by consensus in the same manner as the original recommendations. The panel can also choose at any time to add submitted protocols to the open-access site [19] provided they are in line with the current version of the recommendations.

## Discussion

This paper reports a process designed to generate consensus-based technical recommendations on measuring renal MRI biomarkers. The process has been developed in the context of renal MRI, but is not specific to this application area and could therefore be applied to other potential MRI biomarkers.

The process was developed iteratively and was shaped through the application to four renal MRI techniques with very different issues as far as consensus formation is concerned: *T*_1_ and *T*_2_ contrast is widely used for morphological measures, but quantitative mapping for body applications (to assess fibrosis and oedema) remains to be established; BOLD is technically a very similar method (*T*_2_* relaxation time mapping), but because of the association with oxygenation has developed into a distinct sub-speciality within renal MRI; DWI is an established technique but is very versatile and given enough acquisition time can probe a wide range of structural features such as renal fibrosis, cellular (inflammatory or tumorous) infiltration or oedema; ASL is becoming well established in the brain for assessment of grey matter perfusion, but it is relatively novel in the kidney and product sequences for body ASL are still evolving and have not been settled by the vendors.

Despite these widely different methods, the process proved effective and generated 166 consensus statements in total, with 36 on *T*_1_ and *T*_2_ mapping (17 respondents), 14 on BOLD (24 respondents), 57 on DWI (21 respondents) and 59 on ASL (23 respondents). Combined, it can be expected that these will promote a significant alignment of the research in this area and form the foundation for an international reference standard in clinical renal MRI. These first recommendations should be seen as part of a dynamic process continuously moving towards ever-closer alignment when novel evidence or technologies emerge in the literature.

The areas where no consensus was possible are informative and serve to highlight key open issues that should be prioritised in future research. An interesting example is patient preparation. In the Aarhus meeting, it was agreed that all panels would ask the same questions regarding the need to control diet, hydration status and salt intake before the scan, as the same patient preparation is useful for multiparametric studies. All panels reached consensus on hydration status, but only the BOLD panel had consensus on diet, and only the DWI panel had consensus on salt intake. The results illustrate that the effects on diet and salt intake on most renal MRI biomarkers are not well understood and should be investigated systematically, e.g. by comparing results with different preparation states in the same subjects.

To ensure wider acceptance by the field as a whole, it is critical that the consensus is built by a representative collection of experts. Considering that renal MRI is currently a relatively small field of research, the process has proven effective in generating momentum and critical mass in the response (17–24 respondents per survey). The entire process from defining the scope of the panels until submission of the publications has taken approximately 1 year. This is a relatively fast turnaround time considering the scale of the initiative and the fact that the process itself had not yet been tried and tested.

Interestingly, we found that none of the vendor-specific protocols contributed by various sites in stage 4 of the process were in full compliance with the recommendations made. This by itself provides strong evidence that the end result represents the view of the entire community rather than a small number of authoritative voices. The implication was that no detailed protocols in vendor-specific terminology were able to be uploaded as supplementary material at this stage [19].

### Sustainability and governance

A currently unresolved point of discussion is how to sustain the initiative in the long term. Long-term maintenance is essential in the fast-moving field of MRI physics, but requires a stable, sustainable and well-resourced governance structure. The PARENCHIMA task force will continue to govern the recommendations until the end of the project (May 2021), but it is currently unclear how the programme will progress beyond that.

A good practice example in a related field is the RECIST [23] (Response Evaluation Criteria in Solid Tumours) Working Group funded and governed by the European Organisation for Research and Treatment of Cancer (EORTC). Its mission is “to ensure that RECIST undergoes continued testing, validation and updating” [24]. The working group has created several updates and modifications since the standard was first introduced over a decade ago. Another potential model is the series of “Acute Stroke Imaging Research Roadmaps” [25, 26]. These appear to be maintained and updated in a more ad hoc manner by pairing short meetings of the group with other relevant meetings. The recommendations for ASL in the brain [16] are developed and maintained in a similar way. They were originally developed as part of another COST Action project (ASL in Dementia, BM1103) and through an ISMRM-sponsored workshop. Recently, the community organised a workshop to discuss the need for upgrading, but ultimately decided against this as the field had not sufficiently evolved. Inspiration can also be drawn from the approach to managing expert recommendations on radiological reporting, such as the long-standing Reporting and Data Systems (RAD) maintained by the American College of Radiology (ACR) [27].

For the PARENCHIMA recommendations, a number of avenues were explored, but require further investigation.

The subject matter falls under the remit of the Quantitative Imaging Biomarkers Alliance (QIBA) of the Radiological Society of North America (RSNA), but QIBA has adopted a different evidence-based approach to support the development and confirmation of profiles focused on specific biomarkers. This is opposed to the more general recommendations here, which focus on multiple contrast mechanisms and biomarkers within four renal MRI modalities. Nevertheless, specific biomarkers that emerge from the PARENCHIMA process could potentially be advanced using the QIBA approach. Another consortium funded by the NIH, the Quantitative Imaging Network (QIN), supports development of quantitative imaging tools for the particular application of predicting tumour response to therapy, and also enshrines core values of standardisation, repeatability, and wide translation. Should the PARENCHIMA effort expand towards oncology (renal masses), some collaboration with QIN could be considered.

The governing committee of the Quantitative MR (QMR) study group of the ISMRM was approached, but it does not have a charter of sufficient longevity, nor the resources, to support the maintenance and update of recommendations. The creation of an imaging working group within the European Renal Association was explored with the ERA-EDTA leadership but this did not align well with their organisation around clinical areas. Creating a separate society on renal MRI is a theoretical possibility and the experience of the SCMR (Society of Cardiovascular Magnetic Resonance) has demonstrated that this can be an effective vehicle for development and maintenance of expert recommendations. However, it is doubtful whether the renal MRI field currently has sufficient critical mass to move in that direction. A potential intermediate avenue may be the creation of an ISMRM study group on renal MRI and including maintenance of the recommendations in its mission statement. Potentially, this could be embedded in a broader initiative by the ISMRM to develop and maintain recommendations for MRI data acquisition and analysis across application domains.

### Future developments

Apart from maintenance of the current recommendations, there is a need to develop consensus in other renal MRI biomarkers. Examples of relatively mature areas that would benefit from recommendations are renal dynamic contrast-enhanced MRI or MRI renography [28–34], phase-contrast MRI of the renal arteries [35–38], or MRI volumetry [39–42]. Emerging methods such as magnetisation transfer imaging [43, 44], renal MR elastography [45–48], renal MRI spectroscopy [49, 50], positron emission tomography (PET)/MRI [51], chemical exchange saturation transfer (CEST) [52], 7T renal MRI [53], ^23^Na MRI [54] and hyperpolarised [1-^13^C]pyruvate MRI [55] and ^129^Xe MRI could also be potential candidates for recommendations. Other perspectives that could be taken in future work include the development of recommendations for biomarker panels where different complementary multiparametric sequences are run in the same study, or dedicated recommendations for cross-cutting issues such as region of interest (ROI) definition.

If sufficient capacity can be found, it would be useful to expand the remit of the expert panels to also include field testing of the recommendation. This will involve the supervision and subsequent evaluation of the recommended protocols and collect reporting bias and repeatability coefficients from participating sites. This, in combination with a meta-analysis, can help estimate statistical power for future clinical research studies by interested stakeholders (e.g. new sites or pharmaceutical companies).

A second role that could be added to the remit of the panels, or else be realised through separate programmes [6], is to collect and provide reference data that can help qualify local implementations of the recommended methods. This can include example data on phantoms or healthy volunteer scans of different body compositions with repeatability data. These can then be used as benchmarks to verify implementations in new sites and check for artefacts caused by, for instance, hardware issues, field inhomogeneity, or imperfect shimming.

These roles can build on lessons learnt in neuroimaging, where reproducibility is a very active field of research and a number of best practices for data analysis and data sharing have been recommended recently [56]. Notably, the widely used and simple standard for data organisation “Brain Imaging Data Structure (BIDS) [57]”, makes it much easier to share data, process data and re-run analyses automatically. The BIDS standard could be adapted to other application areas with few modifications.

## Conclusion

The PARENCHIMA process for developing technical recommendations in renal MRI has been developed, reshaped and optimised to achieve a successful application to five very different MRI techniques (ASL, DWI, BOLD, *T*_1_ and *T*_2_). The process is fit for the purpose, having produced 166 recommendations that are widely supported and are likely to promote a more harmonised approach to renal MRI biomarker measurement. In the longer term, we expect this will lead to data that are more directly comparable between sites, scale up the evidence level for clinical utility, and lower the barriers to integrating MRI biomarkers in clinical research.
